# Ecological assessment and environmental niche modelling of Himalayan rhubarb (*Rheum webbianum* Royle) in northwest Himalaya

**DOI:** 10.1371/journal.pone.0259345

**Published:** 2021-11-18

**Authors:** Ishfaq Ahmad Wani, Susheel Verma, Priyanka Kumari, Bipin Charles, Maha J. Hashim, Hamed A. El-Serehy

**Affiliations:** 1 Department of Botany, Conservation and Molecular Biology Laboratory, Baba Ghulam Shah Badshah University Rajouri, Jammu and Kashmir, India; 2 Institute of Biodiversity and Conservation (IBCT), Bangalore, Karnataka, India; 3 Department of Bioscience, University of Nottinghamshire, Nottingham, United Kingdom; 4 Department of Zoology, College of Science, King Saud University, Riyadh, Saudi Arabia; University of Delhi, INDIA

## Abstract

In an era of anthropocene, threatened and endemic species with small population sizes and habitat specialists experience a greater global conservation concern in view of being at higher risk of extinction. Predicting and plotting appropriate potential habitats for such species is a rational method for monitoring and restoring their dwindling populations in expected territories. Ecological niche modelling (ENM) coalesces species existence sites with environmental raster layers to construct models that describe possible distributions of plant species. The present study is aimed to study the potential distribution and cultivation hotspots for reintroducing the high value, vulnerable medicinal herb (*Rheum webbianum*) in the Union territories of Jammu and Kashmir and Ladakh using population attributes and ecological niche modelling approach. Sixty-three populations inventoried from twenty-eight areas display a significant change in the phytosociological attributes on account of various anthropogenic threats. The current potential habitats coincide with actual distribution records and the mean value of Area Under Curve (AUC) was 0.98 and the line of predicted omission was almost adjacent to omission in training samples, thus validating a robustness of the model. The potential habitat suitability map based on the current climatic conditions predicted a total of 103760 km^2^ as suitable area for the growth of *Rheum webbianum*. Under the future climatic conditions, there is a significant reduction in the habitat suitability ranging from -78531.34 Km^2^ (RCP 4.5 for 2050) to -77325.81 (RCP 8.5 for 2070). Furthermore, there is a slight increase in the suitable habitats under future climatic conditions, ranging from +21.99 Km^2^ under RCP 8.5 (2050) to +3.14 Km^2^ under RCP 4.5 (2070). The Jackknife tests indicated Precipitation of Driest Month (BIO14) as the most contributing climatic variable in governing the distribution of *R*. *webbianum*. Therefore, scientifically sound management strategies are urgently needed to save whatever populations are left *in-situ* to protect this species from getting extinct. Present results can be used by conservationists for mitigating the biodiversity decline and exploring undocumented populations of *R*. *webbianum* on one hand and by policymakers in implementing the policy of conservation of species with specific habitat requirements by launching species recovery programmes in future on the other.

## Introduction

*Rheum webbianum* Royle, commonly known as “Himalayan Rhubarb” is an important, vulnerable medicinal plant belonging to family Polygonaceae [1]. It is endemic to Himalayan Biodiversity hotspots covering major parts of India, China, Pakistan, Nepal and Bhutan. In India it occurs in Jammu & Kashmir, Ladakh, Himachal Pradesh, Uttrakhand and is mainly confined to alpine regions ranging between the elevations of 2,400–4,300 m.a.s.l. [2]. The use of *R*. *webbianum* in treating various ailments is as old as human civilization. Both roots and leaves of this species are medicinally important as they are used for the treatment of indigestion, abdominal disorders, boils, wounds, gastritis etc. [3]. In senile patients, its salap is used to boost memory [4]. It shows anti-microbial, antioxidant and anti-diabetic properties [5, 6], besides showing anti-cancerous potential [7]. Owing to its high demand in traditional medicinal practices and pharmaceutical sector, this herb is facing multiple anthropogenic threats in the wild.

Habitat degradation, land-use transformation, unrestrained illegal practices, human exploitation for therapeutic use and inadequate conservation efforts of medicinal plants have plunged them to the verge of extinction [8]. Such interventions have contributed to the near extinction of one fifth of plant species [9]. The rapid rate and magnitude of climate change is one of the dominant factor that leads to the range shift and reduction in the suitable areas for the species which show smaller populations and specific habitat locales [10–13]. Climate change effects are experienced by all types of ecosystems and species, but the Himalayan ecosystems are highly vulnerable to natural hazards, that lead to raising concerns about climate change impacts on the biodiversity of these regions [14–16]. According to different model-based estimates of climate change impacts on plant diversity, mountain ecosystems are among the most susceptible of all terrestrial ecosystems [17–19]. On account of global warming and changes in precipitation pattern, appropriate habitats for several high-altitude plant species could be severely altered or vanished by the end of twenty-first century [20–22]. As a result, it has been proposed that the application of distribution models to determine the extent of species occurrence should be the central concept of different biodiversity assessment and conservation schemes [23, 24]. In order to evaluate the impact of climate change on species distribution, Representative Curve Pathways (RCPs) which determine the probable emission of greenhouse gases and air pollutants in the atmosphere must be considered for different time scenarios (RCP 4.5 and 8.5 for 2050 and 2070) to provide trajectories for climate change [25, 26].

Predicting the boundaries of suitable ecological niches for a species survival forms a baseline in ecology and conservation as it helps in localizing the vital regions that may either require conservation intervention or protection [27]. Reintroduction of the species can be a flourishing ecological engineering approach for strengthening species with reduced populations, their devastated niches, and ecosystems [28–35]. Reintroducing and rehabilitating threatened species require comprehensive information regarding the availability of suitable habitats. Cultivation and restoration of degraded habitats could prove an important and sensible ecological tool for rehabilitation and conservation of threatened plants [33–35]. In conservation management, modelling of species distribution can help to identify areas for rehabilitation and redistribution of threatened medicinal plant species [36–41].

Environmental factors play a significant role in governing the distribution of plant species [42–45]. Ecological niche models of a species integrate bioclimatic variables with occurrence data and represent it on a map that shows the probable distribution of the species [46–48]. These models also present potential habitat suitability under future climatic scenarios which indicate where a particular species has declined or increased. Therefore, making use of ecological niche models is of vital importance to comprehend different environmental factors that determine worthy niche localization for different plant species [49]. Ecological niche modelling can evaluate the link between species occurrence records and the characteristics of the native environment [38]. The occurrence points of the species can be determined via herbaria, direct field observations, and museum-based datasets [50, 51]. The maximum entropy (MaxEnt) utilizes entropy as a measure to extrapolate precise locations of a species presence, and makes it unnecessary to incorporate absence points lying under the conceptual basis [52]. The MaxEnt model is an important ecological modelling statistical tool to help determine possible suitable habitats and potential distribution areas for plant species [53, 54]. MaxEnt has been shown to perform better than other modelling procedures and it requires the “presence only” data. MaxEnt is based on algorithm that recognizes the overall impact of environmental constraints on probable dispersion and dissemination of a particular species [55]. MaxEnt modelling is preferred because it requires only information regarding the geographical coordinates and environmental variables [56, 57] in determining the interactions among the different variables and utilizes species existence locations and environmental variables (categorical and continuous data) for studies pertaining to any particular area [55].

Appropriate ecological principles such as phytosociological analysis and environmental niche modelling are important for the maintenance and conservation of natural populations of threatened plant species. Without knowing the population status, habitat distribution and climatic preference of *R*. *webbianum* it is quite a difficult task to devise practical measures and management strategies to conserve, cultivate or reintroduce this vulnerable medicinal herb. In order to find new perspectives for the conservation of natural territories and resource utilization of this threatened medicinal plant, the present study, aimed to address:

1. Phytosociological analysis and use of recorded geographical coordinates in MaxEnt for ecological niche modelling;
2. Develop a habitat suitability map and predicting suitable habitats for reintroduction and conservation under current climatic scenarios;
3. To determine the area change analysis under future climatic conditions (RCPs 4.5 and 8.5 for two time periods i.e., 2050 and 2070);
4. Identifying the role of different environmental and topographic variables in governing the habitat suitability through jackknife-based analysis;
5. Extensive field surveys and inventories to determine the population status in model predicted niches and relate it to model thresholds.

## Materials and methods

### Target species and study area

**Fig 1:**
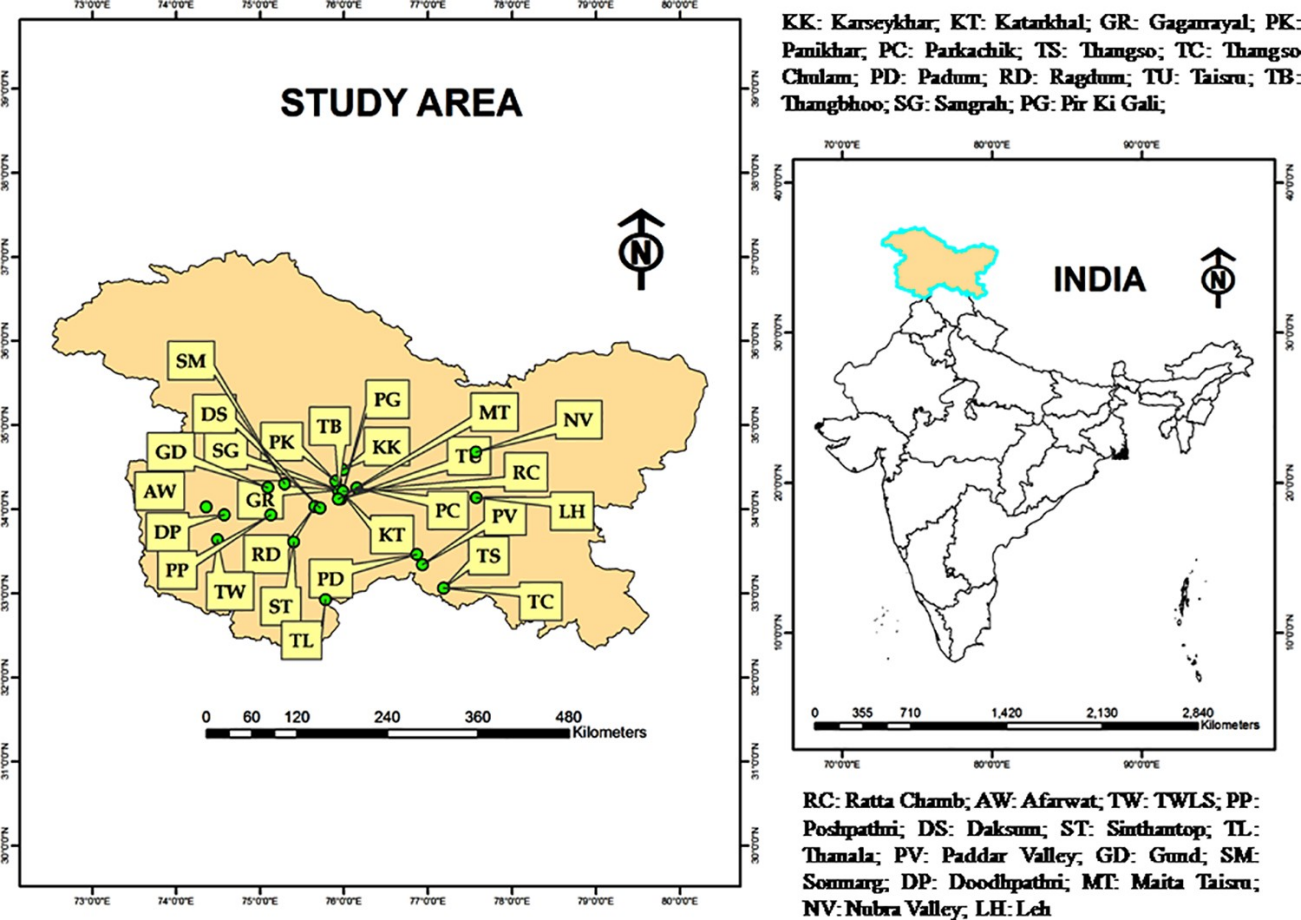
Map showing distribution of different study sites.

*Rheum webbianum* (Polygonaceae) is a perennial herb, inhabiting sub-alpine to alpine regions of Indian Himalaya. It is a highly valued medicinal herb attaining a height of 1.3 to 2.4 meters and bearing thick and fleshy radical leaves. The species overcome cold winters by perrenating underground rhizomes and start their life cycle (germinate) with the advance of favorable climatic conditions. Flowering starts in June, and may extend up to the second week of July. Plants bear numerous notched fruits that hang with thin pedicles. The study was undertaken in alpine and sub-alpine zones of Pirpanjal and Zanskar Himalayan ranges of Jammu and Kashmir and Ladakh UTs from May 2015 to September 2019 (**Fig 1**).

### Vegetation sampling and data collection

Random visits were performed and 63 new populations were located (pre and post modelling) from 28 different areas between the altitudes of 2836 and 4497 m.a.s.l. Geographical coordinates, altitude, and aspects of all the study areas were recorded at the point of observation by Magellan Professional Mobile Mapper (990603–50) and Magellan eXplorist 350H. These occurrence points were then used as a representative habitat for *R*. *webbianum* and subjected to further analysis. Phytosociological analysis was performed by randomly laying an equal number (30) of quadrants (1m^2^) at each study site. Vertical transect method was followed for vegetation sampling [58, 59]. Quantitative analytical characteristics such as density, frequency, abundance, and species distribution patterns were calculated following Curtis and McIntosh [60]. Cluster analysis for all the study sites was performed on the basis of phytosociological characteristics (density, frequency, abundance, distribution pattern) and altitudinal gradients by comparing their Euclidean distances in IBM SPSS20.

### Occurrence and climatic data

Geographical coordinates (latitude and longitude) of *R*. *webbianum* were recorded from the field with a hand-held global positioning system (Magellan Professional Mobile Mapper (990603–50) and Magellan eXplorist 350H. The coordinates were subjected to decimal degree conversion in CSV format (used as a MaxEnt input file) and used in modelling of potential suitable habitats of the species. Nineteen bioclimatic variables (that are highly important in determining eco-physical forbearance of the species) with 30 arc seconds spatial resolution data (that is easily accessible and was obtained from Worldclime dataset v1.4 (https://www.worldclim.org) was used for modelling possible habitat dissemination of the target species [61]. Shuttle Radar Topography Mission Digital Elevation Model data obtained from (http://srtm.usgs.gov/index.php) was used as the source for elevation and slope. All these 21 variables had a spatial resolution of 30 arc seconds (approx. ~ 1 km resolution at the equator).

Global Resource Information Database is the built-in configuration of these extracted files. To make them compatible for the MaxEnt run, these files were transformed to “ASCII” by using “raster to ascii” command in Arc GIS 10.7.1. [50]. Downloaded climatic bio-variables were extracted for the retained occurrence records by using “extract by mask” command.

### Modelling technique and validation

As the occurrence records are most often biased towards geographically convenient and easily accessible areas like cities or areas with higher population density [62]. This results in sampling bias in geographical space [63]. Therefore, to remove this sort of spatial autocorrelation and sampling bias, we used PCA analysis for studying the heterogeneity of different climatic variables of the study area. A map of climatic heterogeneity was then created by combining the three principal component axis using software program SDMtoolbox [64]. By spatial filtering the occurrence records, aggregation among the co-occurrence points was subsequently reduced. After reducing the multiple occurrence points within a single cell, a total of 41 georeferenced points remained as a final dataset for modelling the distribution of *R*. *webbianum*. The process was reproduced 10 times applying bootstrap and calculating the mean and range values [27]. We set the Maximum number of iterations at 5000 and the number of background points were set at 10000. A logistic output was set and a bias grid was created by calculating the Gaussian Kernel density of the sampling sites, using SDMtool box [65].

The use of minimum training presence logistic threshold in model building is suitable for the endangered species as it can predict the larger areas of their distribution range [66]. Besides the use of this logistic threshold in determining the habitat suitability is more appropriate; when the over estimation of the suitable habitats is considered to be less lethal as compared to under estimation [67].

### Environmental (bioclimatic) variables and their exploration

The contribution of different variables is anticipated at training and testing stages of a model run, where each independent variable signifies its gain with respect to other variables in the overall model run. In order to determine the significance of different variables, jackknife experiments were performed ruling out each variable in turn and models were designed with a variable in isolation. The contributions emerge rank-based and deal with the order of predictions. Pearson’s correlation coefficient was determined between different environmental variables. Variables which show clustering and show correlation (r) greater than 0.75 were simplified to only one variable that give most importance to the model output. After correlation analysis, a total of eight variables were selected for modelling the distribution of *R*. *webbianum* under current climate conditions (**Table 1**).

**Table 1:** Pearson’s correlation analysis for different bioclimatic and topographic variables (supplementary file).

	aspect	alt	bio_1	bio_2	bio_3	bio_4	bio_5	bio_6	bio_7	bio_8	bio_9	bio_10	bio_11	bio_12	bio_13	bio_14	bio_15	bio_16	bio_17	bio_18
aspect	1.00																			
alt	**-0.13**																			
bio_1	**0.13**	**-0.94**																		
bio_2	**0.05**	**0.34**	**-0.17**																	
bio_3	**0.01**	**0.30**	**-0.12**	**0.90**																
bio_4	**0.09**	**-0.44**	**0.31**	**-0.52**	**-0.81**															
bio_5	**0.15**	**-0.94**	**0.98**	**-0.15**	**-0.19**	**0.47**														
bio_6	**0.10**	**-0.91**	**0.98**	**-0.18**	**-0.03**	**0.13**	**0.91**													
bio_7	**0.10**	**-0.07**	**0.01**	**0.05**	**-0.38**	**0.80**	**0.22**	**-0.21**												
bio_8	**0.05**	**0.43**	**-0.48**	**0.20**	**0.37**	**-0.52**	**-0.54**	**-0.36**	**-0.42**											
bio_9	**0.13**	**-0.89**	**0.94**	**-0.27**	**-0.21**	**0.35**	**0.92**	**0.91**	**0.02**	**-0.47**										
bio_10	**0.14**	**-0.95**	**0.99**	**-0.24**	**-0.24**	**0.47**	**0.99**	**0.93**	**0.16**	**-0.53**	**0.93**									
bio_11	**0.11**	**-0.87**	**0.97**	**-0.04**	**0.10**	**0.06**	**0.90**	**0.99**	**-0.20**	**-0.36**	**0.89**	**0.91**								
bio_12	**-0.03**	**-0.75**	**0.75**	**-0.49**	**-0.27**	**0.13**	**0.66**	**0.82**	**-0.36**	**-0.17**	**0.68**	**0.72**	**0.75**							
bio_13	**0.03**	**-0.81**	**0.82**	**-0.41**	**-0.29**	**0.28**	**0.77**	**0.84**	**-0.15**	**-0.24**	**0.75**	**0.81**	**0.79**	**0.96**						
bio_14	**-0.01**	**-0.70**	**0.72**	**-0.37**	**-0.10**	**-0.04**	**0.60**	**0.82**	**-0.49**	**-0.04**	**0.62**	**0.66**	**0.76**	**0.97**	**0.91**					
bio_15	**-0.04**	**-0.26**	**0.28**	**0.24**	**-0.09**	**0.51**	**0.42**	**0.13**	**0.68**	**-0.29**	**0.18**	**0.37**	**0.16**	**0.09**	**0.29**	**-0.02**				
bio_16	**0.01**	**-0.80**	**0.81**	**-0.43**	**-0.26**	**0.22**	**0.74**	**0.85**	**-0.23**	**-0.19**	**0.73**	**0.79**	**0.79**	**0.98**	**0.99**	**0.95**	**0.21**			
bio_17	**0.02**	**-0.75**	**0.76**	**-0.40**	**-0.14**	**0.03**	**0.65**	**0.85**	**-0.44**	**-0.08**	**0.68**	**0.71**	**0.79**	**0.98**	**0.94**	**0.99**	**0.00**	**0.97**		
bio_18	**-0.15**	**-0.42**	**0.43**	**-0.46**	**-0.18**	**-0.13**	**0.30**	**0.55**	**-0.58**	**0.05**	**0.36**	**0.37**	**0.48**	**0.90**	**0.79**	**0.88**	**-0.06**	**0.82**	**0.86**	
bio_19	**0.06**	**-0.86**	**0.88**	**-0.46**	**-0.32**	**0.31**	**0.82**	**0.89**	**-0.16**	**-0.32**	**0.82**	**0.86**	**0.84**	**0.95**	**0.98**	**0.89**	**0.21**	**0.98**	**0.93**	**0.73**

### Model evaluation

A set of the different feature combinations (Linear, Product, Quadratic, Threshold and Hinge) was developed for MaxEnt models with different complexities for Regularization Multipliers values ranging from 0.5–2. The model complexity is reduced by increased values of RM by managing the figure of variables entering the model [55]. For future projections, ENMveal package in R software was used to select ideal feature and RM values [68]. Finally, on the basis of omission rates the models were ranked and the best model was chosen on lowest 10% emission and 0% test emission **(Table 2)**

**Table 2:** Summary of model evaluation statistics of *Rheum webbianum* using ENMveal with varying model complexities.

S.No	Maxent Features	Variables	RM	AUCcv	Test OR 0%	Test OR 10%	Rank
1	LH	Alt, Aspect, Bio1, Bio2, Bio8, Bio-4, Bio14, Bio15	1	0.9886	0.025	0.19	8
2	LH		1.5	0.9885	0.025	0.16	7
3	LH		2	0.9874	0.02	0.11	1
4	LQH		1	0.9891	0.085	0.15	15
5	LQH		1.5	0.9873	0.045	0.09	10
6	LQH		2	0.9884	0.04	0.11	9
7	LQP		1	0.9882	0.02	0.135	2
8	LQP		1.5	0.9888	0.025	0.105	5
9	LQP		2	0.9871	0.025	0.145	6
10	LQPT		1	0.9896	0.05	0.165	13
11	LQPT		1.5	0.9898	0.02	0.135	3
12	LQPT		2	0.9893	0.02	0.155	4
13	LQPTH		1	0.9908	0.05	0.14	12
14	LQPTH		1.5	0.9895	0.075	0.12	14
15	LQPTH		2	0.9899	0.05	0.125	11

L, Q, P, T, H represent linear, product, threshold and hinge features in Maxent; RM: Regularization Multiplier; AUCcv represents the mean 25-fold cross validated models developed during evaluation process; Test OR represents the omission rates at 0% and 10%, respectively.

### Niche modelling for current period

To model the current distribution of *R*. *webbianum* we used freely available MaxEnt software (Maximum Entropy Distribution), version 3.4.1 (http://www.cs.princeton.edu/~schapire/maxent/), which provides an approximate likelihood of occurrence of the species. We used the Area under the curve (AUC) of the receiver operating characteristics (ROC) to evaluate model performance [64]. AUC values range from 0–1 with AUC value between 0.5 and 0.7 representing poor model performance, 0.7–0.9 indicating good performance, and > 0.9 showing high performance [52]. The model was run ten times and the average AUC values were calculated. Further, ArcGIS 10.7.1 was used for classifying the final map into 2 classes: suitable and not suitable.

In order to select the best model for *R*. *webbianum* we used ENMveal package in R software [70]. We used 10 fold cross validation and AUCcv to create the binary maps of suitable and unsuitable areas. We used 10% training presence cloglog threshold. The value used is 0.5134. All the areas between 0 and 0.5314 is considered as absent while areas with greater than 0.5314 is considered as present [69–71].

### Future climatic projections

In order to develop future niche models, we used the same set of climatic variables that were used to model the current distribution pattern of *R*. *webbianum*. To predict the future potential distribution and impact on the climate change on the distribution patter of *R*. *webbianum*, Hadley Global Environment Model 2-Earth System (HADGEM2-ES) representing simulations for two representative concentration pathways (RCP4.5 and RCP8.5) were obtained from the fifth assessment report (AR5) of the Intergovernmental Panel for Climate Change (IPCC) [72]. RCPs use a variety of radiative forcing to model different GHG concentration trajectories. The RCP 4.5 emission scenario is optimistic with emissions peaking about 2050 and then declining over the next 30 years. The RCP 8.5 represents the pessimistic scenario as it anticipates the emission of greenhouse gases throughout the twenty-first century. We created the models for two time periods 2050s (2040–2069) and 2080s (2070–2099) for both RCPs. We categorized the final model output into two suitability categories as: Not suitable and suitable. Using DIVA-GIS [61] (www.diva-gis.org), we calculated the percentage of area change by simple differencing.

### Population status in relation to model thresholds

In order to corroborate the validation and relevance of the model thresholds associated with the population status of the species in each occurrence locality, large-scale field surveys, and inventories were performed. The numerical strength of the plant species (density) at different localities was tallied with the threshold levels (very high, high, medium, and low) in the distribution models. Populations bearing greater densities were superimposed with higher thresholds to confirm habitat suitability for reintroduction of species and vice versa [73].

### Habitat status assessment for species reintroduction

**Fig 2:**
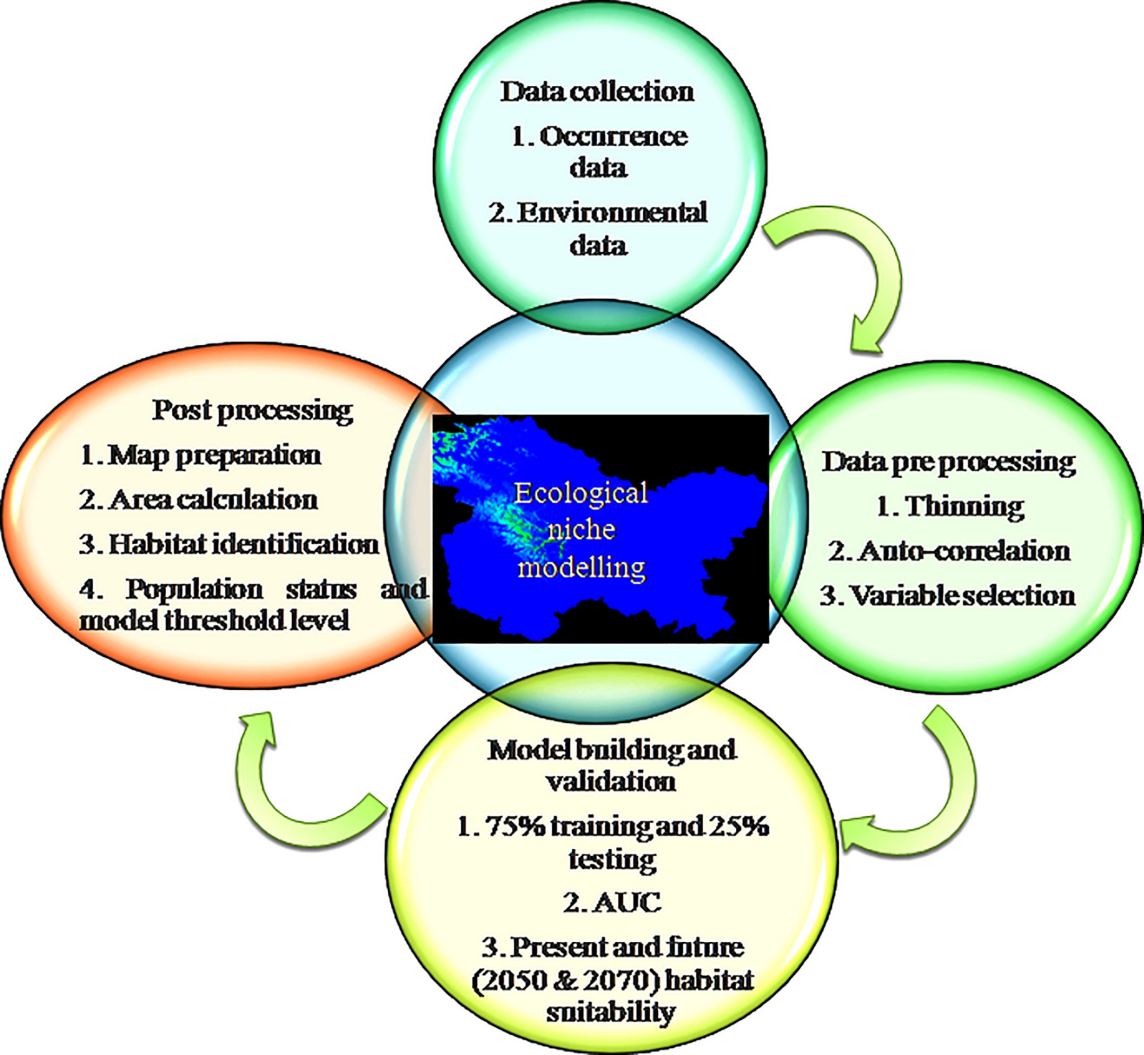
Flow chart of methodology applied for determining the habitat suitability of *R*. *webbianum*.

Niche suitability prediction maps were changed to KMZ format using Diva GIS ver. 7.3 (www.diva-gis.org) and then overlaid on Google Earth Ver. 6 (www.google.com/earth) images for assessing the actual habitat condition prevailing in the areas of occurrence. On the basis of model output, repeated field surveys were carried out in the entire predicted potential area to assess actual habitat suitability. The methodology applied in determining the habitat suitability of *R*. *webbianum* is shown in **Fig 2**.

## Results

### Vegetation sampling and quantitative analysis

**Fig 3:**
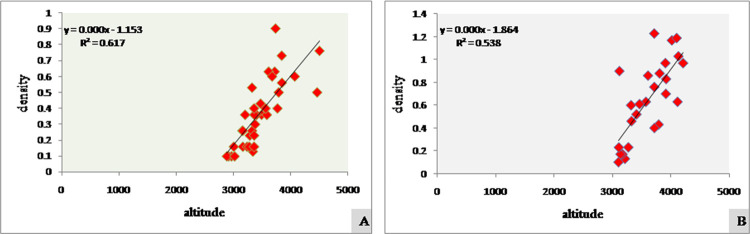
Correlation between altitude and density at, A) study areas which were used for MaxEnt analysis, B) study areas which were predicted as possible suitable areas by MaxEnt run.

During the entire study period, 37 new populations were reported (pre-modelling) from 16 different areas of the Pirpanjal and Zanskar mountain ranges of Indian Himalaya. The density of *R*. *webbianum* ranged between 0.1 ind/m^2^ (Sangrah 5) and 0.9 ind/m^2^ (Panikhar) while frequency ranged between 6% (Panikhar 5) and 70% (Sinthantop 1). The abundance to frequency (A/F) ratio of *R*. *webbianum* revealed that a greater number of the populations (16) show a random distribution pattern. Thirteen populations exhibit a contagious type of distribution (occur in clusters) while eight populations were regularly distributed. Species from the Pirpanjal range mainly prefer facing the South-East slope while species from the Zanskar mountain regions face toward the North-East (NE) and East (E) directions. The populations inhabit subalpine to alpine regions within elevations ranging from 2889 m.a.s.l. to 4497 m.a.s.l., and population attributes such as density and frequency increased with an increase in elevation. Populations that lie below an altitudinal range of 3000 m. a. s. l. had the lowest densities and were recognized as the least suitable habitats. Elevations between 3000–3500 m.a.s.l. were designated as moderately suitable habitats while elevations greater than 3500 m.a.s.l. showed greater density of *R*. *webbianum* and were the reported to be the best suitable habitats. Detailed phytosociological analysis is provided in **Table 3**. A strong positive Pearson’s correlation coefficient was reported between altitude and density (**Fig 3**).

**Table 3:** Phytosociological attributes and habitat suitability thresholds of *R*. *webbianum* at Pirpanjal and Zanskar Himalayan ranges (sites used as training and test locations).

S. No	Population Site	Habitat	Al.	As.	Coordinates	H.S.T	D	F	A	A/F
**1**	Karseykhar 1	Rocky	3488	NE	34° 14.856’N	M	0.5	36.7%	1.36	0.037
					75° 59.858’E					
**2**	Karseykhar 2	Bouldery	3841	NE	34° 14.620’N	H	0.73	63.3%	1.15	0.018
					75° 59.946’E					
**3**	Karseykhar 3	Exposed bushy	3287	NE	34° 14.759’N	M	0.46	33.3%	1.75	0.131
					75° 59.366’E					
**4**	Karseykhar 4	Exposed dry	3341	NE	34° 14.620’N	M	0.43	30%	1.33	0.133
					75° 59.646’E					
**5**	Katarkhal 1	Exposed moist	3474	SE	34 ^o^13^/^43.721^o^N	M	0.43	33.3%	1.3	0.039
					75 ^o^57^/^02.912^o^E					
**6**	Katarkhal 2	Exposed bushy	3809	SE	34 ^o^13^/^43.811^o^N 75 ^o^57^/^02.682^o^E	H	0.76	56.7%	1.17	0.059
**7**	Garagrayal	Rocky	3767	SE	34 ^o^13^/^43.731^o^N	H	0.73	30%	1.22	0.040
					75 ^o^57^/^02.482^o^E					
**8**	Panikhar 1	Bouldery	4067	NE	34° 10.605’N	H	0.6	43.3%	1.38	0.031
					75° 54.363’E					
**9**	Panikhar 2	Bouldery	3327	NE	34°10.692’N	M	0.26	16.7%	1.6	0.095
					75° 54.439’E					
**10**	Panikhar 3	Exposed moist	3734	NE	34° 10.710’N	H	0.9	70%	1.28	0.01
					75° 55.027’E					
**11**	Panikhar 4	Exposed dry	3491	NE	34° 10.655’N	M	0.36	26.7%	1.37	0.05
					75° 54.297’E					
**12**	Parkachik1	Rocky moist	3206	NE	34° 05.626’N	M	0.36	26.7%	1.37	0.05
					76° 09.069’E					
**13**	Parkachik 2	Exposed moist	3718	NE	34° 05.192’N 76° 10.588’E	H	0.63	43.3%	1.46	0.03
**14**	Parkachik 3	Exposed moist	3767	NE	34° 03.197’N	M	0.4	30%	1.33	0.044
					76° 12.787’E					
**15**	Parkachik 4	Bouldery moist	3845	NE	34° 02.764’N 76° 14.515’E	H	0.56	50%	1.13	0.022
**16**	Thangso 1	Bouldery	4497	E	33° 04.256’N 77° 11.409’E	H	0.76	56.7%	1.35	0.023
**17**	Thangso 2	Exposed moist	3456	E	33° 04.396’N	M	0.4	40%	1.25	0.031
					77° 11.518’E					
**18**	Thangso Chulam	Exposed moist	3167	E	33° 04.592’N 77°11.413’E	L	0.16	13.3%	1.25	0.093
**19**	Padum (Obra) 1	Exposed moist	3240	E	33°28.169’N	L	0.16	13.3%	1.25	0.093
					76°52.568’E					
**20**	Padum (Obra) 2	Exposed moist	3158	E	33° 27.960’N	M	0.34	13.3%	2	0.150
					76° 52.963’E					
**21**	Padum (Obra) 3	Exposed dry	3583	E	33°27.058’N 76°51.248’E	M	0.36	30%	1.22	0.040
**22**	Padum (Obra) 4	Exposed dry	3614	E	33°26.651’N 76°50.561’E	H	0.63	40%	1.58	0.039
**23**	Parkachik 5	Exposed moist	3378.8	E	34^o^04^’^21.75^o^N	M	0.3	23.3%	1.28	0.054
					75^o^49^’^32.110^o^E					
**24**	Ragdum	Bouldery	3404.3	N.E	34^o^02^’^11.11^o^N	M	0.36	26.6%	1.37	0.051
					75^o^39^’^10.017^o^E					
**25**	Taisuru	Rocky mountain	2976.9	N.E	34^o^07^’^22.788^o^N	L	0.16	13.3%	1.25	0.093
					75^o^57^’^22.746^o^E					
**26**	Thanghboo	Rocky Mountain	3264.7	E	34^o^13^’^05.514^o^N75^o^56^’^10.386^o^E	M	0.36	23.3%	1.14	0.048
**27**	Sangrah1	Rocky Mountain	3062.9	E	34^o^12^/^47.646^o^N75 ^o^59^/^02.976^o^E	L	0.23	23.3%	1	0.042
**28**	Sangrah2	Rocky Mountain	3561.2	E	34 ^o^12^/^47.647^o^N	M	0.4	40%	1	0.025
					75 ^o^59^/^02.975^o^E					
**29**	Sangrah3	Moist bushy	3673.7	N.E	34 ^o^12^/^47.634^o^N	H	0.6	30%	1.8	0.042
					75 ^o^59^/^02.987^o^E					
**30**	Sangrah4	Moist exposed	3368.8	N.E	34 ^o^12^/^47.638^o^N	M	0.36	30%	1.1	0.036
					75 ^o^59^/^02.992^o^E					
**31**	Sangrah5	Moist bushy	2969.2	N.E	34 ^o^12^/^47.637^o^N 75 ^o^59^/^02.992^o^E	L	0.1	6%	1.5	0.25
**32**	Pir Ki Gali	Dry exposed	3361.4	N.E	34 ^o^12^/^47.647^o^N 75 ^o^59^/^02.976^o^E	M	0.4	30%	1.33	0.044
**33**	Pir Ki Gali	Moist bushy	3319	E	34 ^o^12^/^47.621^o^N	M	0.39	36%	1.5	0.25
					75 ^o^59^/^02.952^o^E					
**34**	Ratta Chamb1	Rocky mountain	3161.4	SE	34 ^o^12^/^47.648^o^N	M	0.36	33.3%	1.2	0.09
					75 ^o^59^/^02.974^o^E					
**35**	Ratta Chamb2	Moist bushy	3389	SE	34 ^o^12^/^47.648^o^N	M	0.4	40%	1	0.25
					75 ^o^59^/^02.954^o^E					
**36**	Afarwat1	Moist Sandy	3326	SE	34 ^o^01^/^47.238^o^N	M	0.53	30%	1.6	0.053
					74 ^o^21^/^26.766^o^E					
**37**	Afarwat2	Moist bushy	3723.3	SE	34 ^o^01^/^38.952^o^N	H	0.76	43.4	1.77	0.040
					74 ^o^21^/^21.888^o^E					

Al. Altitude; As. Aspect; H.S.T. Habitat Suitability Threshold; D. Density; F: Frequency; A. Abundance; M. Medium; H. High; L. Low.

### Model building and validation

Six bioclimatic variables i.e., Annual Mean Temperature (bio-1), Mean Diurnal Range (bio-2), Temperature Seasonality (bio-4), Mean Temperature of Wettest Quarter (bio-8), Precipitation of the driest month (bio-14), Precipitation Seasonality (bio-15) and two topographic variables Elevation (Elv.) Aspect (Asp.) were selected. After the multi-collinearity test, we made sure that the selected set of predictors has a VIF values less than 5. The final selected model has a combination of LH (Linear, and Hinge features) and RM value of 2 and AUCcv = 0.9874. During the tuning experiments all the models performed better than random.

**Fig 4:**
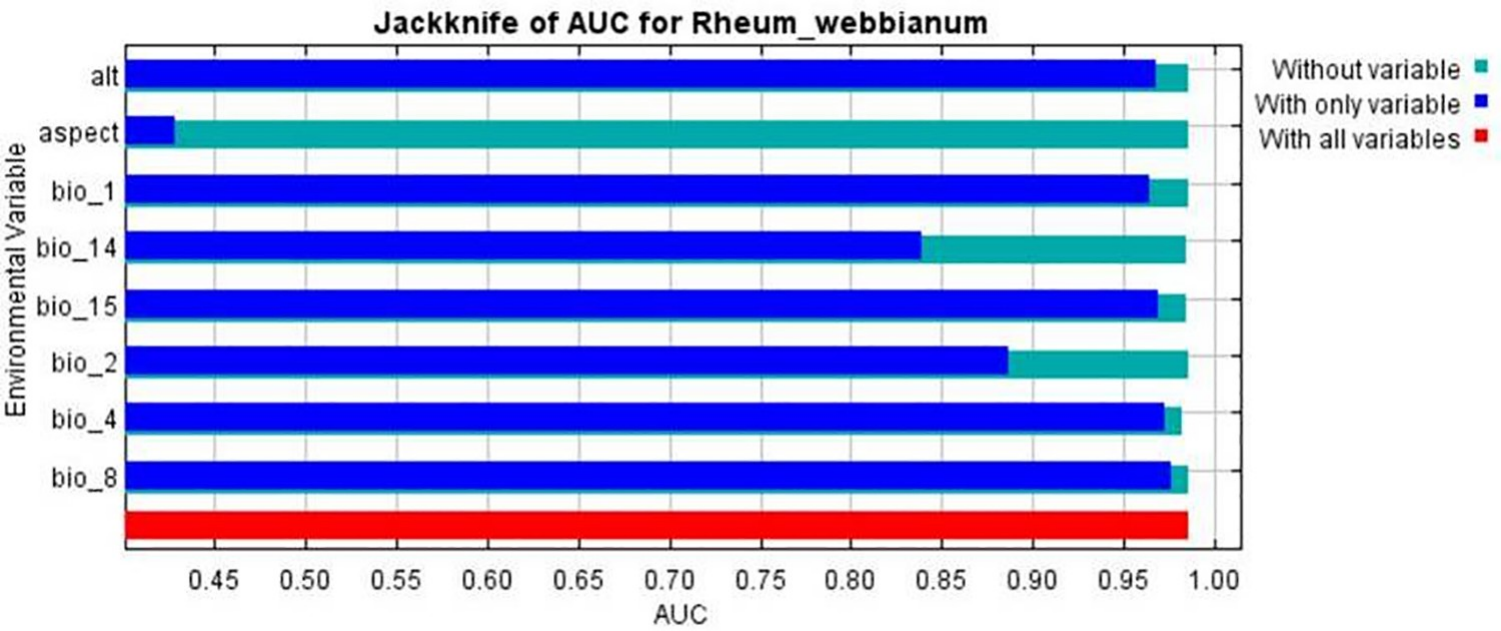
Jackknife results for contribution of different environmental and topographic variables.

Based on the results of the Jack-knife test, Precipitation of Driest Month (bio-14) was the most contributing climatic variable in governing the distribution of *R*. *webbianum* with a percent contribution of 32.8, followed by Temperature Seasonality (standard deviation *100) (bio-4) (22% contribution) and Precipitation Seasonality (Coefficient of Variation) (bio-15) (16.4% contribution). However, aspect was the least effect variable with a percent contribution of 2.2%. (**Table 4; Fig 4**).

**Table 4:** List of the environmental variables used in modelling after Pearson’s multi-collinearity test.

Variable	Description	Temporal scale	Percent contribution
bio-1	Annual Mean Temperature	Annual	7.7
bio-2	Mean Diurnal Range	Variation	11.1
bio-4	Temperature Seasonality	Variation	22
bio-8	Mean Temperature of Wettest Quarter	Quarter	7.3
bio-14	Precipitation of Driest Month	Month	32.2
bio-15	Precipitation Seasonality	Variation	16.4
Elv.	Elevation	Topographic	1.1
Asp.	Aspect	Topographic	2.2

Source: Hijmans et al. [61]

**Fig 5:**
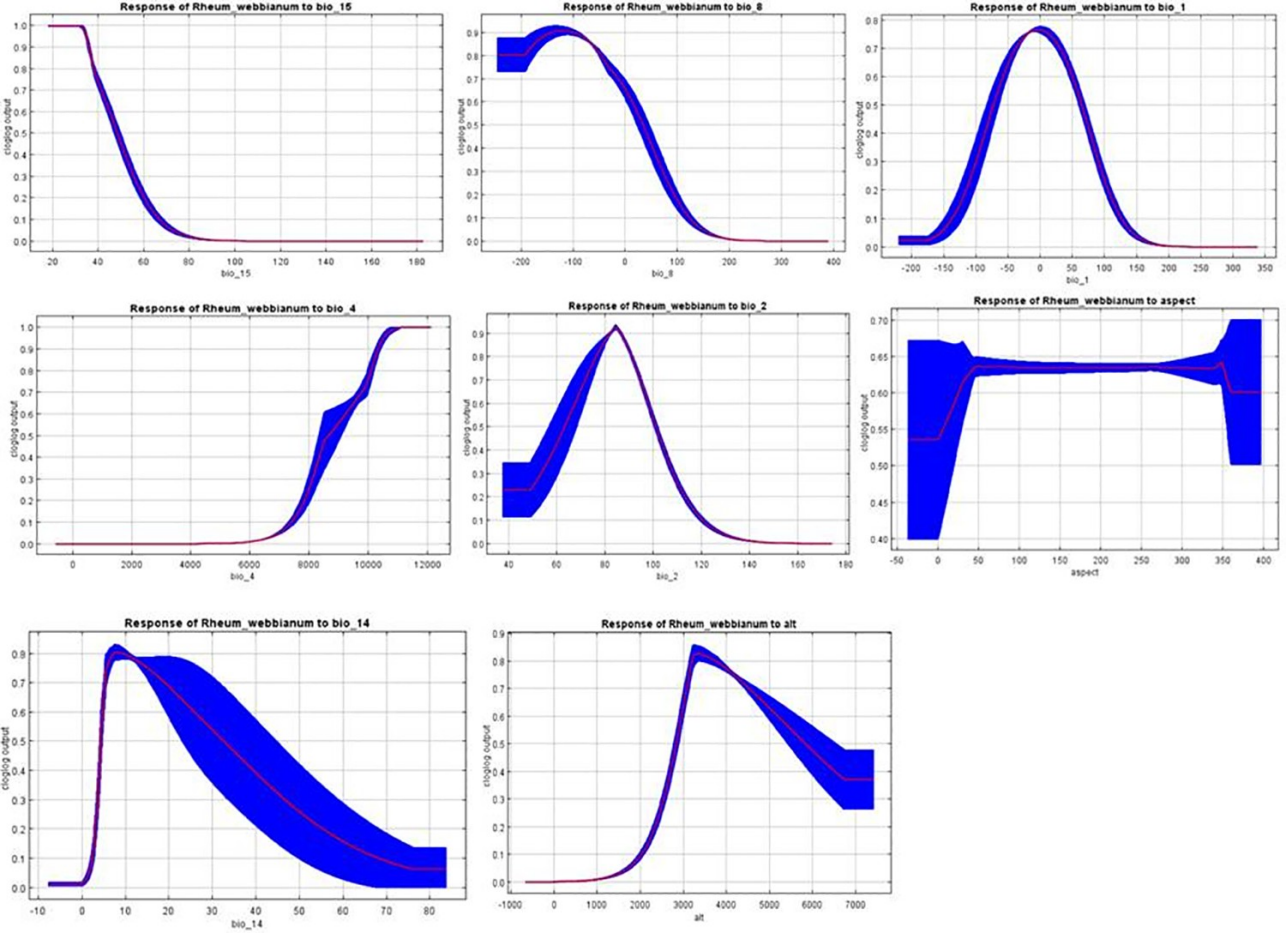
Response curves showing dependence of habitat suitability on the selected variables.

Response curves produced by the MaxEnt depict the influence of different variables on the probability predictions. Response curves display the consequence of logistic predictions as these variables are modified while other variables are kept at their average value. However, such curves do not demonstrate the potential for multi-variant interactions that can be employed within the model. The logistic output for *R*. *webbianum* peaked towards the higher value of bio-4. The probabilities peaked around the low values of bio-8 and bio15. The habitat suitability of *R*. *webbianum* showed a gradual increase with bio-4 while increased values of bio-15, bio-14 and bio-8. The habitat suitability for *R*. *webbianum* increased upto certain altitudes (**Fig 5**).

### Current habitat distribution

Cumulative threshold means that omission rate and predicated omission should be close to each other. While these attributes are the functions of the cumulative threshold, the omission rate is determined by training presence and test records. In (**S1 Fig)** the black line indicates predicted emission, the red line indicates fraction of background predicted (mean area), and the blue line indicates omission on training samples. The line of predicted omission is very close to omission in training samples. Mean value of AUC (area under ROC) curves obtained while developing a habitat suitability model was 0.986 i.e. close to 1, indicating that the model run was fairly accurate (**S2 Fig**).

**Fig 6:**
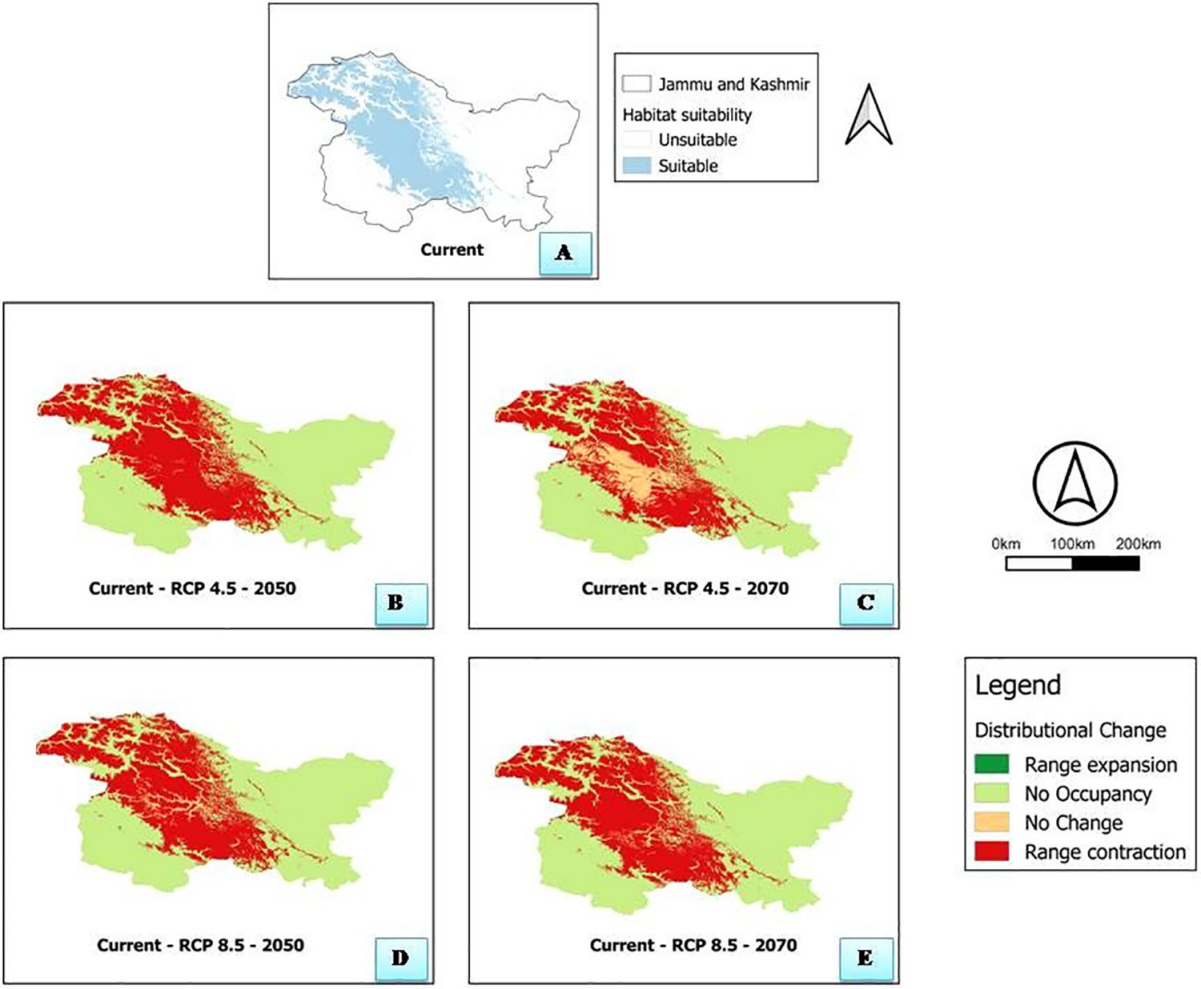
**A:** Current suitable and unsuitable habitats of *Rheum webbianum* **B and C:** Species range change under RCP 4.5 for 2050 & 2070 **D and E:** RCP 8.5 for 2050 & 2080.

Areas that could form potential habitats with greater suitability thresholds were disseminated to the higher altitudes of Pirpanjal and Zanskar sub ranges of the Northern Himalaya of India. Under the current climatic conditions, the suitable areas for growth of *R*. *webbianum* occurs mainly in North-eastern and central parts of Kashmir valley including most of Srinagar, Ganderbal, Bandipora, Anantnag, Budgam and Northern part of Kulgam and Eastern part of Pulwama. Western areas of Rajouri and Poonch, Northern parts of Doda and entire of Kishtwar in Jammu province; entire of Kargil and south western parts of Leh in Ladakh province represent areas suitable for the growth of *R*. *webbianum* under current climatic conditions. (**Fig 6A**).

### Species range dynamics under future climatic scenarios

The results based on the future climatic scenarios (RCPs 4.5 and 8.5) for two time periods (2050 and 2070), predicted a large decrease in the overall habitat suitability for *R*. *webbianum*. In general, the model showed extreme decrease in the overall future habitat suitability ranges between under RCP 4.5 (2050) to and RCP 8.5 for 2070. A moderate habitat contraction was seen under RCPs 8.5 for 2050. 60% of the suitable habitats are predicted to be lost in these future scenarios **(Table 5)**.

**Table 5:** Total suitable areas under current and future climatic scenarios for two time periods.

Output Key	Current—RCP 4.5 2050	Current—RCP 4.5 2070	Current–RCP 8.5 2050	Current—RCP 8.5 2070
Range expansion	-	3.14	21.99	3.14
No Change	220.69	3,102.17	13,538.04	1,426.21
Range contraction	78,531.34	75,649.85	65,213.98	77,325.81

The current suitable areas that becomes non-suitable for its growth in the future includes central and major parts of Anantnag, Kulgam and Pulwama in Kashmir valley, majority of Kargil and Leh. However, the only areas that remain suitable under future conditions include, some parts of Srinagar, Baramulla and Ganderbal in Kashmir and Northern part of Kargil in Ladakh except under RCP 8.5 2070 where the suitable habitats will be found only in Northern Kargil. In some areas of Kishtwar and Ganderbal, we also observed a slight increase in the suitable habitats for RCP 8.5 (2050) (**Fig 6B–6E**).

### Population status in relation to model thresholds and identification of areas for reintroduction

A total of 489 individuals were inventoried in areas recognized as potential suitable habitats. Of these, 79% of populations (recognized from field observations before modelling) and 69% of populations (recognized from field observations after modelling) represent imbalanced population structure, i.e., seedling, sapling, young, and adult individuals were poorly represented or absent. Populations such as Karesykhar 2, Katarkhal 2, Garagrayal, Panikhar 1,3, Parkachik, 2,4, Thangsoo 1, Padum 4, Sangrah 3, Afarwat 2, and TWLS 1, Daksum2, Sinthantop1, Sonmarg, Maita Taisuru1, Nubra 1, Leh,1, and Panikhar 1showed a greater degree of regeneration within a balanced population structure. All these localities present a better habitat suitability threshold level for *R*. *webbianum* and fall under greater threshold categories. These populations account for 30.15% of the total populations, followed by medium (50.81%) and low thresholds (19.04%). These observations confirm the strong correlation between population size and level of model thresholds that show a high degree of relatedness to these findings.

Superimposing the predicted potential habitat map of the species on Google Earth satellite images suggested that the plant exhibits patchy presence within alpine to subalpine zones of mountain peaks running through the Pirpanjal (Rajouri, Poonch, Shopian and Pulwama, Anantnag, Baderwah, Ganderbal) and Zanskar regions (Kishtwar, Kargil, and Leh). Combined efforts from post modelling field surveys, Google Earth satellite images and model output showed that areas with high to very high habitat suitability for the species were rocky mountain peaks and exposed moist areas, while medium to low habitat suitability was reported for sub alpine zones, exposed moist and bushy areas, human civilized areas, and hillocks. All these results were supported by the population attributes of the plant species at these areas. Detailed phytosociological analysis is provided in **Table 6**.

**Table 6:** Phytosociological attributes of *R*. *webbianum* at different sites of Pirpanjal and Zanskar Himalayan ranges (sites recognized on ENM analysis).

S. No	Population Site	Habitat	Alt.	As.	Coordinates	H.S.T	D	F	A	A/F
**1**	TWLS1	Bouldery	4132	S.W	34 ^o^ 12^/^47.64^o^ N	H	1.03	56.6	1.82	0.032
					75 ^o^ 59^/^02.96^o^ E					
**2**	TWLS2	Exposed bushy	3321	S.W	34 ^o^ 12^/^47.67^o^ N 75 ^o^ 59^/^02.96^o^ E	M	0.53	50	1.67	0.033
**3**	Gagarrayal	Exposed moist	3573	N.E	33°8726’N 74°5754’E	M	0.63	43.4	1.46	0.033
**4**	Poshpathri	Rocky moist	2972	E	33°.9342’ N 75°.1137’ E	L	0.23	16.7	1.4	0.083
**5**	Daksum1	Rocky	3193	E	33°.6114’ N 75°.4359’ E	M	0.43	20	2.16	0.108
**6**	Daksum 2	Rocky	3719.98	N.W	33°.6114’ N 75°.4359’ E	H	0.4	26.7	1.5	0.056
**7**	Sinthantop1	Bouldery	4213	N.W	33°.5811’ N 75°.5102’ E	H	0.97	70	1.39	0.019
**8**	Sinthantop 2	Exposed dry	3019	N.E	33°.5811’ N 75°.5102’ E	L	0.17	13.3	6%	1.5
**9**	Thanala	Exposed moist	2914	S.W	32°.9227’ N 75°.7735’ E	L	0.13	13.3	1	0.075
**10**	Nalthi	Exposed bushy	3003	S.W	32°.9227’ N 75°.7735’ E	L	0.10	13.3	1	0.075
**11**	Machail	Rocky moist	2993	N.W	33°.3913’ N 76°.3695’ E	L	0.17	10	1.67	0.167
**12**	Paddar Valley	Exposed bushy	2836	N.W	33°.45.681’N 76°.17.911’E	L	0.17	10	1.67	0.167
**13**	Gund 1	Moist bouldery	3009	S.E	34°17′21″N 74°48′45″E	L	0.23	10	2.33	0.233
**14**	Gund 2	Exposed dry	3326	S.E	34°17′21″N 74°48′45″E	M	0.46	30	1.56	0.052
**15**	Sonmarg	Exposed moist	3717.78	.E	34°17′21″N 74°48′45″E	H	0.76	43.4	1.77	0.040
**16**	Doodhpathri	Bouldery	3118.19	E	33.8510° N 74.5635° E	M	0.9	53.4	1.68	0.031
**17**	Doodhpathri	Bouldery	3318	N.E	33.8510° N 74.5635° E	M	0.6	36.7	1.64	0.044
**18**	Maita Taisuru1	Rocky mountain	3612	N.E	34° 07’30.366’N	H	0.76	43.4	1.77	0.040
					75° 55’53.670’E					
**19**	Maita Taisuru2	Rocky mountain	3464	S.E	34° 07’30.476’N	M	0.36	30%	1.22	0.040
					75° 55’53.783’E					
**20**	Nubra valley 1	Moist exposed	3917.19	S.E	34.6863° N 77.5673° E	H	0.7	43.4	1.61	0.037
**21**	Nubra Valley 2	Rocky moist	3247	S.W	34.6863° N 77.5673° E	M	M	0.43	20	2.16
**22**	Leh 1	Exposed dry	3718	N.E	34.1383° N 77.5727° E	H	1.23	80	1.54	0.019
**23**	Leh 2	Exposed dry	3310	N.E	34.1383° N 77.5727° E	M	0.86	50	1.74	0.034
**24**	Panikhar 1	Exposed bushy	3774	NE	34° 10.817’N	H	0.97	70	1.39	0.019
					75° 55.307’E					
**25**	Panikhar 2	Exposed bushy	3312	NE	34° 10.110’N	M	0.43	20	2.16	0.108
					75° 55.017’E					
**26**	Panikhar 3	Exposed bushy	3374	NE	34° 10.210’N	M	0.43	20	2.16	0.108
					75° 55.097’E					

Abbreviations: Alt: Altitude, As: Aspect, HST: Habitat Suitability Threshold, D: Density, F: Frequency, A: Abundance, TWLS: Tatakooti Wildlife Sanctuary, SW: South West, NE: North East, E: East, H: High, M: Medium, L: Low.

**Fig 7:**
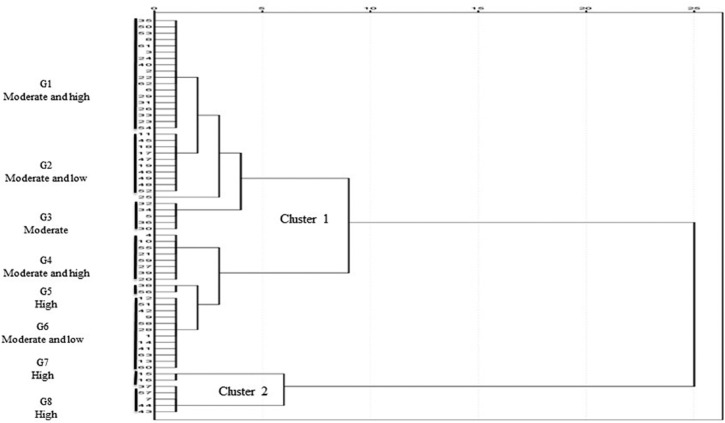
Cluster analysis of different populations showing range of habitat relatedness in northwestern Himalaya (Jammu & Kashmir and Ladakh UT’s) of India.

To understand the degree of habitat relatedness of *R*. *webbianum* in Pirpanjal and Zanskar ranges (Jammu & Kashmir and Ladakh UTs) cluster analysis was performed by IBM SPSS, 20. The results of clustering revealed that the 63 populations could be broadly classified into two clusters. The major cluster contained 6 groups with 56 populations while the minor cluster held 2 groups and 7 populations. Five of the eight groups had related populations with similar habitat suitability while the others contain populations with varying suitability ranges (**Fig 7**). Population 25, which falls under cluster1, was distinctly related to the other groups. These observations could be related to phytosociological analysis (density, frequency, abundance and distribution pattern), altitudinal gradients, model map, and threshold levels with a greater degree of appreciation (**Tables 3 and 6**).

## Discussion

In the era of Anthropocene, increased global temperatures and alterations in the precipitation pattern [74], tends to modify the habitat and distribution of endemic plant species thereby increasing the risk for extinction [75, 76]. The species with unprecedented anthropogenic threats, narrow distribution range, smaller population structure and greater habitat specificity or included under the IUCN category of threat could be highly vulnerable to alterations of their distribution range and to degradation or loss of their habitat in near future [77–80]. Based on the direct field observations and phytosociological analysis, current pattern of population structure of *R*. *webbianum* confirms all the above- mentioned parameters for *R*. *webbianum*, besides confirming its distribution to higher elevations (2900–4500 m.a.s.l.). Restricted distribution of *R*. *webbianum* shows that it is endemic to the Himalaya where alpine zones form its best habitat. Available literature on its distribution have revealed that this plant species has restricted distribution within alpine or sub alpine zones of Himalaya [81].

Predicting suitable ecological niches under current and future climatic scenarios is a critical approach for management and protection of threatened and endemic species [82, 83]. The actual niche of the species is generally smaller as compared to the area predicted through model-based predictions, because the climatic variables are not only sole determinants of habitat suitability [84]. Different biotic and edaphic factors act as limiting variables and provide a subtle role to govern the habitat distribution of a species [85]. On the other hand, it is of utmost significance to point out that when niche modelling is performed for greater geographical areas, climate is usually regarded as the most significant driver of species occurrence [86, 87]. Our findings reveal that temperature-based variables gained higher values as compared to precipitation-based variables for predicating the distribution of *D*. *hatagirea*. Greater preference to temperature based climatic parameters indicated that this medicinal plant respond significantly to these and are important in structuring the distribution of *D*. *hatagirea*. This finding is equally supported by the results of other workers [88–90] who also modelled the dominant role of temperature related climatic variables in governing the distribution of medicinal plants in the north western Himalaya. Variability of precipitation pattern also acts as a significant parameter that influences the species distribution in the Himalaya [91]. Heavy snowfall in the Western Himalaya as compared to Eastern Himalaya may contribute to increased occurrences of these plant species compared to eastern Himalaya. These findings are in accordance with the observations of [92] who have suggested that the distribution pattern of Himalayan alpine species is governed by precipitation variability and long-lasting snow cover.

Determining the role of different topographic and environmental variables in shaping and maintaining the species distribution range is a critical issue in ecology and evolution. These variables propose logical classifications on the subtle role of environmental agents to ascertain the niche suitability of a species [93]. Environmental gradients like climate, soil, and geology have credible impact on different vegetation indices in a particular area [94] that are quite evident in the temporal and spatial variations of different vegetation indices such as EVI and NDVI. The greater support of bioclimatic variables such as BIO 14, BIO4 and BIO 15 to the overall niche example echoes the subtle part played by such constraints for explaining habitat appropriateness in case of *R*. *webbianum*. Importantly, these variables were not only beneficial to the habitat suitability model, but also agreed with the term of their different phenophases. Therefore, EVIs have the ability to act as potent and informatory substitute variables that can depict the ambiguous formulations of the underlying environmental agents that establish the peripheries of the capable niche/ habitat of the species [73].

The predicted potential distribution of *R*. *webbianum* was in agreement with our infield observations; the plant species showing major habitats towards alpine peaks. These findings are in accordance with the results of Tayade et al. and Rashid et al. [4, 95] who also explored this plant from the alpines of different areas. Future climatic scenarios produce a profound effect on the distribution range of threatened species [96]. Our results predict that future climatic changes may result in a major decline in the potential distribution of *R*. *webbianum* thereby drastically reducing the habitat suitability. Representative Concentration Pathways (RCP’s) depict the increased concentration of Green House Gases (GHG’s) especially under 8.5 climatic scenarios in comparison to 4.5, thereby resulting in the decline of major habitat range throughout 2070. These predictions are in accordance with the findings of Dullinger et al.; Molloy et al.; Barrett et al.; [96–98] who also reported decline in the habitat suitability of different plant species on account of increased concentration of GHG’s under RCP’s 4.5 & 8.5.

Better population status, which coincides with greater model thresholds of the species under study at higher elevations, depicts the habitat appropriateness for effective persistence of the species. Certain altitudes that were predicted to show better habitat suitability display squeezed population size. Timely field observations revealed different anthropogenic disturbances (trampling and grazing) as a driving force for the population degradation in such cases. Based on these interpretations, we can undertake that population structure of *R*. *webbianum* in unruffled niches within its local range could be affirmed via this model output, i.e., locales accommodating larger population size can be considered as exemplars with raised threshold level and vice versa.

An anthropobiome constitutes an area that provides the favorable conditions for successful establishment of the plant species [99]. Predicated suitable areas of *R*. *webbianum* as explored through direct field surveys, model output, and Google Earth satellite images fall under different suitability threshold levels, i.e., high, medium, and low. In this regard, planning for species reintroduction should proceed carefully. This study suggests that for this purpose, areas such as sub alpine zones, exposed moist and bushy areas, human civilized areas, and hillocks form habitats with a medium to high level of habitat suitability. Areas with sparse populations could be used as habitats for efficient reintroduction provided that the threat posed on the plant species is reduced. Google Earth superimposed with field surveys displayed a similar kind of habitat appropriateness, endorsing the use of Google images in assessing habitat suitability. This assessment approves the application of GES, which can be used effectively as an alternative for broad field inspections [100].

### Future implications for its conservation

In order to avoid unexpected consequences to overall functioning of the ecosystem, reintroduction of the plant species and restoration of their depleted populations must be performed with vigilant considerations. Lessening possible threats that are primary drivers of species endangerment, introduction of the species in a strictly controlled situation might be one of the basic strategies for preserving species with a high risk of extinction. Species distribution modelling approach may act as an important tool to determine the current distribution besides playing a subtle role in determining the effect of climate change on the habitat suitability and species range change (geographic distribution). This may also help in determining the landscape connectivity and provide the much valuable information regarding ecological niches in its complete distribution range. Although future climatic scenarios show a considerable contraction in the habitat suitability, however the limited suitable areas need to be maintained. These areas will act as refugia for the better survival of this plant under future climatic conditions.

Different integrative *in-situ* conservation strategies and use of captive propagation in controlled environments such as natural habitats, botanic gardens, and other conservation facilities could greatly aid in increasing the recovery rate of this vulnerable medicinal herb as well as facilitate in its germplasm conservation. Protecting its populations in the natural habitat and restoring ecosystems requires the combined efforts of various non-government organizations, aboriginals, educational and research institutions, and different government agencies. In this regard, many institutions should work in tandem with direct field observations and host different programs to preserve the overall gene pool of this plant. Establishment of field repositories, bio-banks and cryo-conservation plants for the collection and preservation of specimens and genetic material should be used as a potential *ex situ* strategy for its long-term survival. Use of the advanced biotechnological applications, such as high throughput genotyping and gene sequencing, metabolomics, meta-genomics, and transcriptomics are preferred for genetic characterization of the plant, and may lead to taxonomic and evolutive characterization of this plant. Bioinformatics coupled with the above-mentioned biotechnological tools can allow for the interpretation of genotypic information and add to bio-bank archives. Putting these tools in the hands of researchers and scientists for their successful application on *R*. *webbianum* may represent an important tool to safeguard its gene pool in the future.

## Conclusions

The current study describes the application of ecological niche modelling (ENM) and population attributes to identify the areas that support *R*. *webbianum* populations using sophisticated spatial resolution data, occurrence points, and environmental variables. This study provides the first predicted potential habitat distribution map of *R*. *webbianum* in India’s Northwest Himalaya, which can assist in exploring new populations and developing better land use regulation near the species natural territories. The areas located through current distribution modelling can be used for the re-introduction of *R*. *webbianum*. Under future climatic scenarios (RCP’s 4.5 & 8.5), this plant species shows a drastic decrease in the habitat suitability (27,000 sq. kms.) as compared to the current prediction where the suitable habitats range across 1,03,000 sq. kms. Based on habitat contraction prediction in the near future, potential suitable areas must be prioritized and maintained at an utmost importance. Activities related to anthropogenic disturbances and change in land use patterns such as expansion of human settlements, agricultural activities and livestock production threaten the population structure as revealed by different phytosociological characters. This study helps to categorize the different regions on the basis of habitat suitability, as well as aim focus on the priority to refurbish a species’ natural niche for efficient conservation. The results showed strong familiarity with population structure and model thresholds suggesting the remarkable value of ENM and population studies in devising appropriate conservation strategies. The regions sought for reintroduction of the *R*. *webbianum* will not only lead to ecological improvement in the devastated niches, but also help in the rehabilitation of *R*. *webbianum* populations, and thereby promote its conservation.

## Supporting information

S1 Fig Average omission and predicted area for *Rheum webbianum*. (JPG)

S2 Fig Receiver operating characteristic curve with mean area under curve (AUC) for *R*. *webbianum*. (JPG)
