# Correction: Ecological assessment and environmental niche modelling of Himalayan rhubarb (*Rheum webbianum* Royle) in northwest Himalaya

**DOI:** 10.1371/journal.pone.0261100

**Published:** 2021-12-02

**Authors:** The PLOS ONE Staff

There is an error in the Funding statement. The correct statement is: This study received support from the Department of Biotechnology, Ministry of Science of Science and Technology, Govt. of India, under Research Grant Number BT/Env/BC/01/2010.

There is an error in affiliation 1 for authors Ishfaq Ahmad Wani, Susheel Verma, and Priyanka Kumari. The correct affiliation is: Conservation and Molecular Biology Laboratory, Department of Botany, Baba Ghulam Shah Badshah University Rajouri, Jammu and Kashmir, India.

The publisher apologizes for the errors.

There are errors in the author contributions. The correct contributions are: Funding acquisition: HAES. Methodology: IAW, SV, BC. Project administration: HAES. Software: IAW, SV, BC, MJH. Supervision: SV. Writing – original draft: IAW, SV, PK. Writing – review & editing: SV, IAW, HAES.
