# Supplementary figures and images for: Ecological assessment and environmental niche modelling of Himalayan rhubarb (Rheum webbianum Royle) in northwest Himalaya
Source: PLoS One. 2021 Nov 18;16(11):e0259345. doi: 10.1371/journal.pone.0259345 (PMC8601538; doi:10.1371/journal.pone.0259345)

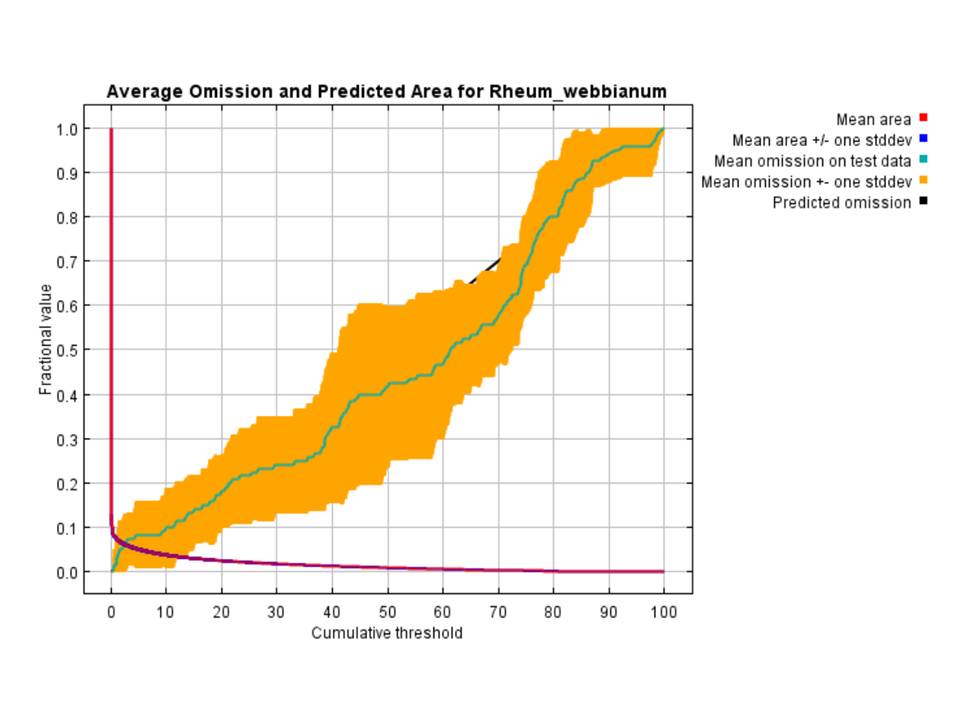

Supplement: S1 Fig — (JPG) [file pone.0259345.s001.jpg]

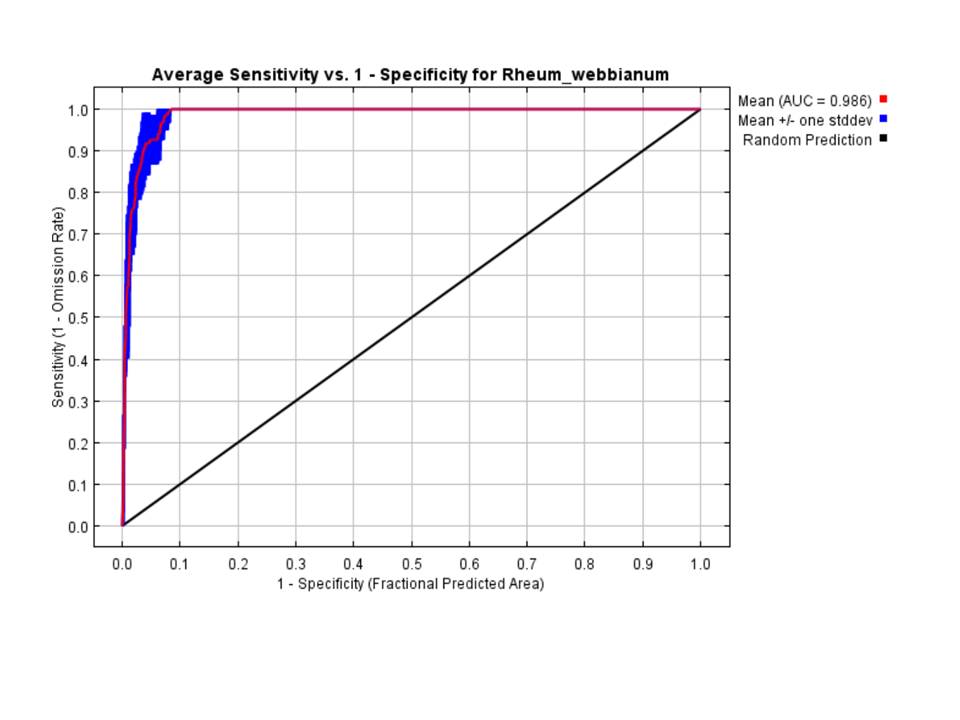

Supplement: S2 Fig — (JPG) [file pone.0259345.s002.jpg]
